# A Neurofibromatosis Noonan Syndrome Patient Presenting with Abnormal External Genitalia

**DOI:** 10.4274/jcrpe.galenos.2019.2019.0023

**Published:** 2020-03-19

**Authors:** Esra Işık, Hüseyin Onay, Tahir Atik, Aslı Ece Solmaz, Samim Özen, Özgür Çoğulu, Şükran Darcan, Ferda Özkınay

**Affiliations:** 1 Ege University Faculty of Medicine, Department of Pediatrics, Division of Pediatric Genetics, İzmir, Turkey; 2 Ege University Faculty of Medicine, Department of Medical Genetics, İzmir, Turkey; 3 Ege University Faculty of Medicine, Department of Pediatrics, Division of Pediatric Endocrinology, İzmir, Turkey

**Keywords:** Neurofibromatosis Noonan syndrome, NF1 gene, abnormal external genitalia

## Abstract

Neurofibromatosis Noonan syndrome (NFNS) is a rare RASopathy syndrome, resulting from *NF1* gene mutations. NFNS is characterized by phenotypic features of both neurofibromatosis type 1 *(NF1)* and Noonan syndrome. Plexiform neurofibromas (PNFs) are an unusual finding in NFNS. A seven year-old girl with typical clinical features of *NF1* was referred to our clinic due to short stature and abnormal genital appearance. Due to dysmorphic features, a clinical diagnosis of NFNS was considered in the patient and, following molecular analysis, revealed a novel heterozygous c.3052_3056delTTAGT (p.L1018X) variant in the *NF1* gene. Although evaluation for genital virilization, including karyotype and hormonal studies were normal, imaging studies revealed a diffuse genital PNF. Although PNFs are seen rarely in NFNS, this should be considered in the differential diagnosis of genital virilization in these patients to prevent unnecessary testing.

**What is already known on this topic?**

The Neurofibromatosis Noonan syndrome (NFNS) is a rare RASopathy syndrome characterized by phenotypic features of both neurofibromatosis type 1 *(NF1)* and NS. It occurs as a result of *NF1* gene mutations. Plexiform neurofibromas (PNFs) are seen rarely in NFNS patients.

**What this study adds?**

A novel mutation in the *NF1* gene in a patient who presented with phenotypic features of both *NF1* and NS is reported. A PNF was present in this case and was responsible for an unusual genital phenotype. Thus PNF may be a rare cause of genital virilization in NFNS patients.

## Introduction

Neurofibromatosis Noonan syndrome (NFNS) (OMIM 601321) is a rare RASopathy syndrome, first defined in four unrelated patients in 1985 by Allanson et al (1). These patients who were originally diagnosed as neurofibromatosis type 1 (NF1), also presented with clinical features of NS. NFNS patients show phenotypic features of both NF1 and NS. In subsequent years several cases showing clinical findings of both syndromes have been reported, thus introducing a new phenotypic syndrome; NFNS. NFNS has been shown to be due to heterozygous mutations in the *NF1* gene (2).

Neurofibromas are benign peripheral nerve sheath tumors and are classified as either dermal or plexiform neurofibromas (PNFs) (3). PNFs are usually congenital and originate from a bundle of fascicles or a large nerve plexus. PNFs can be seen in 25-50% of NF1 patients (4). However, they are more rarely seen in NFNS patients. It has been reported that in pediatric NF1 patients PNFs are most commonly localized in the head and neck region (4,5). Genital localization of PNFs has been reported rarely.

In this study, we report a young girl with NFNS and a genital PNF manifesting as abnormal external genitalia giving the impression of a disorder of sex development (DSD).

## Case Report

A seven year-old girl with typical clinical features of NF1 was referred to our clinic due to short stature and abnormal genital appearance. She was born at the 37^th^ week of gestation with a birth weight and height of 2800 gr (10-25^th^ centile) and 47 cm (10-25^th^ centile), respectively. During the newborn period multiple café-au-lait spots and spina bifida were observed. Her neuromotor development was normal. At the age of three years an operation was performed for filum terminale lipoma. A family history revealed her father had similar clinical features.

**Figure 1:**
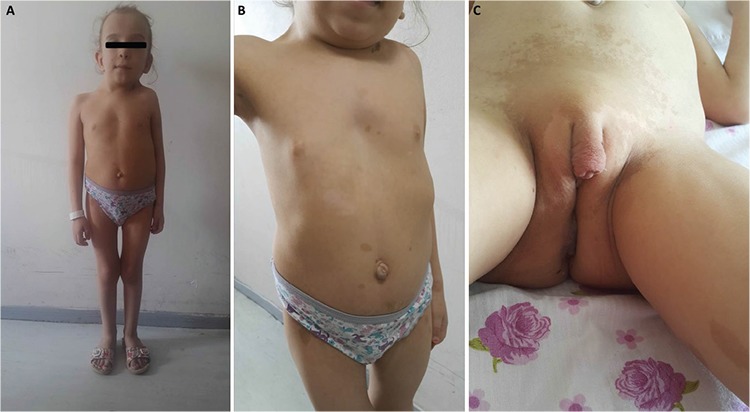
Phenotypic findings of the patient. A, B) Dysmorphic features: macrocephaly, broad forehead, sparse eyebrows, depressed nasal bridge, hypertelorism, low set ears, deeply grooved philtrum, short and webbed neck, pectus excavatum and multiple cafe-au-lait spots. C) Cliteromegaly

On physical examination, weight, height and head circumference were measured as 18 kg (3-10^th^ percentile), 106 cm (<3^rd^ percentile), 54 cm (>97^th^ percentile), respectively. She had macrocephaly, broad forehead, sparse eyebrows, depressed nasal bridge, hypertelorism, low set ears, deeply grooved philtrum, short and webbed neck, pectus excavatum, kyphoscoliosis, sacral hypertrichosis, multiple cafe-au-lait spots and axillary and inguinal freckling (Figure 1). On genital examination, abnormal external genitalia were observed. A genital structure resembling a phallus was measured as 3.5 cm. Genital appearance was evaluated as being Prader Stage 2. No Lisch nodule was detected via slit-lamp examination. Echocardiography was normal. Cranial magnetic resonance imaging (MRI) revealed a hamartoma.

**Figure 2:**
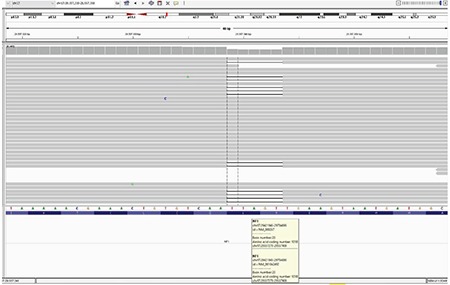
Next generation sequencing analysis in the patient demonstrating a heterozygous *NF1* mutation; c.3052_3056delTTAGT (p.L1018X)

Due to clinical features including macrocephaly, short stature, facial dysmorphism, webbed and short neck and pectus excavatum in addition to the typical findings of NF1, a clinical diagnosis of NFNS was considered in the patient. All coding exons and the flanking intronic regions of the *NF1* (NM_000267.3) and the *PTPN11* (NM_002834) genes were amplified by polymerase chain reaction and sequenced using Illumina MiSeq platform (Illumina Ltd., San Diego, USA). Molecular analysis revealed a novel heterozygous c.3052_3056delTTAGT (p.L1018X) variant in the *NF1* gene (Figure 2). In accordance with the American College of Medical Genetics recommendations (null variant, hot-spot region, variant not found in public databases) this variant has been predicted as pathogenic (6).

**Figure 3:**
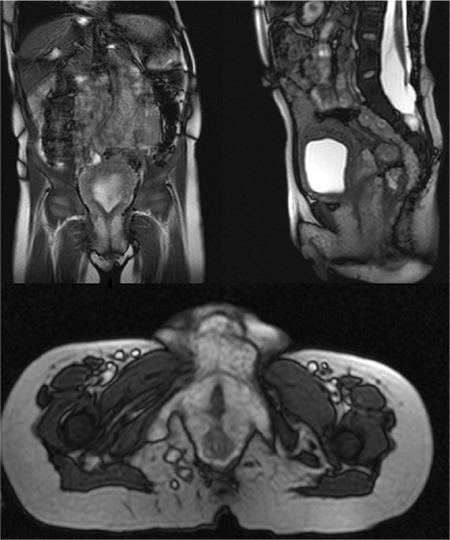
Magnetic resonance imaging of abdomen and external genitalia: multiple plexiform neurofibromas filling the pelvic area with invasion of the sacral neural foraminae and subcutaneous region, multiple neurofibromas in the labium majus and the external genital area

Laboratory tests for genital virilization, including karyotype (46,XX), thyroid function, follicle stimulating hormone, luteinizing hormone, estradiol, testosterone, 17-hydroxyprogesterone, 11-deoxycorticosterone, dehydroepiandrosterone sulphate, adrenocorticotropic hormone and cortisol concentrations and bone age were all normal (Table 1). MRI of the pelvis and external genitalia showed multiple PNFs filling the pelvic area with invasion of the sacral neural foraminae and subcutaneous region. Additionally, multiple neurofibromas were detected in the labium majus and the external genital area (Figure 3).

## Discussion

NS and NF1 are both included within the group of conditions known as the RASopathy syndromes. However, distinct clinical and genetic differences exist. The patient presented here had a number of features typical of both syndromes. NS is genetically heterogeneous. To date at least 14 genes, the most common being *PTPN11*, have been implicated in the etiology of NS (7). NF1 patients have characteristics different to NS while carrying *NF1* gene deletions or point mutations (2,8). To date, a number of cases, showing characteristic cardinal findings of both syndromes together with *NF1* gene mutations have been reported in the literature. Among NFNS patients, several patients were found to have mutations in both *PTPN11* and *NF1*. The majority of NFNS patients, however, have been reported to have only *NF1* mutations, without any detectable *PTPN11* mutation. Thus NFNS syndrome has been attributed to *NF1* mutations by most authors. In our patient, following sequencing of both *NF1* and *PTPN11* genes, the only pathogenic variant found was in *NF1* (9).

In NF1 patients mutations may appear anywhere throughout the entire gene. However, in the majority of NFNS patients, mutations have been localized within the GTPase-activating-protein (GAP) related domain (2). Consistent with this, the novel frameshift mutation observed in our patient was localized in the GAP related domain.

Disorders of sex development are classified into three groups (sex chromosome DSD, 46,XY DSD and 46,XX DSD) based on karyotype (10). In 46,XX DSD the genotype is 46,XX and the gonads present as ovaries; however, the external genitalia shows virilization (11). Virilization of external genitalia is rarely caused anything other than hormonal factors. Due to the abnormal appearance of the external genital organs in this case, a differential diagnosis for DSD was necessary. To exclude 46,XX DSD, hormonal tests were performed and they were all found to be normal. Subsequent imaging studies revealed a diffuse PNF which resulted in clitoral and labial enlargement in the patient. PNFs in external genital organs have rarely been described in NF1 patients (12,13) and to date there have been no reports of NFNS patients with PNFs invading the external genitalia. In the current literature, the hormonal analyses of all NF1 patients with genital abnormality have been found to be normal (12,13,14,15).

Neurofibromas are benign tumors, however their invasive nature and size may require surgical intervention. In almost 50% of PNFs involving the genital area, surgical intervention has been required (4). Unfortunately surgical correction was not an option for our patient due to the extensive involvement of the PNF in the pelvic area and the patient was started on sirolimus treatment, as this was seen as the most appropriate form of treatment.

Firstly, if a patient presents with the clinical features of both NS and NF1, NFNS should be considered. Secondly, in NFNS, as in NF1, genital PNF is an unusual clinical finding and could present as abnormal external genitalia giving the impression of a DSD. Imaging studies, prior to extensive hormonal work-up, should be performed to rule this out.

## Figures and Tables

**Table 1:**
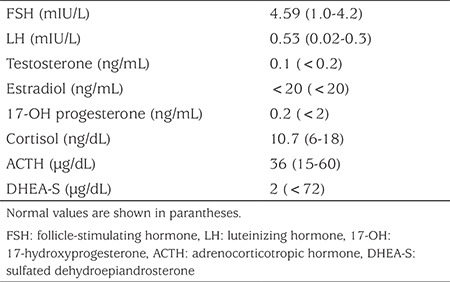
Hormone concentrations of the patient at presentation
